# Association between Coagulation Profile and Clinical Outcome in Children with SARS-CoV-2 Infection or MIS-C: A Multicenter Cross-Sectional Study

**DOI:** 10.3390/children9020279

**Published:** 2022-02-17

**Authors:** Danilo Buonsenso, Francesco Mariani, Luca Pierri, Rosa Morello, Adriana Yock-Corrales, Olguita Del Aguila, Ilaria Lazzareschi, Giuseppe Zampino, Francesco Nunziata, Piero Valentini, Andrea Lo Vecchio

**Affiliations:** 1 Department of Woman and Child Health and Public Health, Fondazione Policlinico Universitario A. Gemelli, 00168 Rome, Italy; ilaria.lazzareschi@policliniclgemelli.it (I.L.); giuseppe.zampino@policlinicogemelli.it (G.Z.); 2 Dipartimento di Scienze Biotecnologiche di Base, Cliniche Intensivologiche e Perioperatorie, Università Cattolica del Sacro Cuore, 00168 Rome, Italy; 3 Global Health Research Institute, Istituto di Igiene, Università Cattolica del Sacro Cuore, 00168 Roma, Italy; 4 Institute of Pediatrics, Università Cattolica del Sacro Cuore, 00168 Roma, Italy; francesco.mariani.100292@gmail.com (F.M.); rosa.morello91@gmail.com (R.M.); 5 Department of Translational Medical Sciences—Section of Pediatrics, University of Naples Federico II, 80131 Naples, Italy; luca.pierri90@gmail.com (L.P.); franc.nunziata@gmail.com (F.N.); 6 Pediatric Emergency Department, Hospital Nacional de Niños “Dr. Carlos Sáenz Herrera”, CCSS, San José 10103, Costa Rica; adriyock@gmail.com; 7 Unidad de Infectología Pediátrica del Hospital Nacional Edgardo Rebagliati Martins, Lima 15072, Peru; odaguila@hotmail.com

**Keywords:** COVID-19, SARS-CoV-2, D-dimers, coagulation, children

## Abstract

Limited data on the coagulation profile in children affected by the SARS-CoV-2 infection are available. We aimed to evaluate the role of d-dimers as predictors of poor outcomes in a pediatric population affected by the SARS-CoV-2 infection or multisystem inflammatory syndrome (MIS-C). We performed a retrospective cross-sectional multicenter study. Data from four different centers were collected. Laboratory tests, when performed, were collected at the time of diagnosis, and 24, 48, 72, 96, 120 and beyond 120 h from diagnosis; blood counts with formula, an international normalized ratio (INR), activated partial thromboplastin time (aPTT), D-dimers and fibrinogen values were collected. Data regarding clinical history, management and outcome of the patients were also collected. Three hundred sixteen patients with a median age of 3.93 years (IQR 0.62–10.7) diagnosed with COVID-19 or MIS-C were enrolled. Fifty-eight patients (18.3%) showed a severe clinical outcome, 13 (4.1%) developed sequelae and 3 (0.9%) died. The univariate analysis showed that age, high D-dimer values, hyperfibrinogenemia, INR and aPTT elongation, and low platelet count were associated with an increased risk of pediatric intensive care unit (PICU) admission (*p* < 0.01). Three multivariate logistic regressions showed that a d-dimer level increase was associated with a higher risk of PICU admission. This study shows that D-dimer values play an important role in predicting the more severe spectrum of the SARS-CoV-2 infection, and was higher also in those that developed sequelae, including long COVID-19.

## 1. Introduction

For about a year and a half now, the world has been facing the most relevant pandemic of the last century. SARS-CoV-2 has spread rapidly since the first case was described in December 2019 [1] and, despite major progresses in the understanding of the coronavirus disease (COVID-19) and the development of effective vaccines, the number of infected people is still increasing [2].

SARS-CoV-2 infection can be associated with a wide clinical variability, ranging from asymptomatic infection [3] to a severe disease characterized by acute severe respiratory syndrome and multisystem involvement that can lead to death [4,5]. Initially, several studies in the adult population described the clinical presentation and laboratory abnormalities in the first phase of the symptomatic disease, focusing on the cytokine storm that characterizes the more severe spectrum of disease. However, it has progressively become clear that coagulation abnormalities play a pivotal role in the pathophysiology of both acute disease and long COVID-19 [6,7].

In the pediatric population, the SARS-CoV-2 infection is usually asymptomatic or associated with mild symptoms [8,9,10]. However, both severe cases and deaths have been reported worldwide, with higher rates in children with severe comorbidities or in those living in Low-to-Middle Income Countries [11]. Moreover, a relatively small percentage of children develops, some weeks after infection, a clinically more severe multisystem inflammatory syndrome (MIS-C) that requires intensive care [12] in up to 68% of cases. Although the laboratory signature of an acute SARS-CoV-2 infection in children is relatively well described, being characterized by either normal findings or lymphopenia, an increase in D-dimers, C-reactive protein [13,14], and the MIS-C has a known cardiologic involvement characterized by also an increase in troponin and proBNP [12]. The coagulation profile of the more severe spectrum of the SARS-CoV-2 infection—including MIS-C—is relatively poorly studied in children, since most studies mainly focused on clinical and demographic data. Taking into account the role that disorders of coagulation play in adults with COVID-19, we hypothesized that children with a severe SARS-CoV-2 infection might present similar alterations. Therefore, we performed a multicenter study to analyze a cohort of patients with an acute SARS-CoV-2 infection or diagnosed with MIS-C to evaluate the predictors of severe disease, with a specific focus on the role of coagulation biomarkers.

## 2. Materials and Methods

A multicenter cross-sectional study involving patients from four different academic hospitals (two in Italy, one in Costa Rica and one in Perù) was conducted in the period between March 2020 and August 2021. The two Italian centers enrolled all consecutive pediatric patients, younger than 18 years of age, diagnosed with COVID-19, ascertained by a positive RT-PCR on nasopharyngeal swab, or diagnosed with MIS-C, in accordance with the World Health Organization (WHO) indications [15], either admitted to the hospital or discharged by the emergency department. Conversely, the two Latin American centers only enrolled all consecutive MIS-C patients or children with COVID-19 that required admissions. This decision was based on the possibility of Latin American centers to perform blood tests, including coagulation profile, only in the most ill children. These four centers have been selected as part of the COVID-DOMINGO network, which already published several studies in the field of pediatric COIVD-19 [16,17,18,19,20]. Epidemiological, demographic, clinical, laboratory data and information concerning treatment and clinical outcomes were collected by an operator in each hospital and entered into an electronic database built on purpose for the present study and subsequently used for statistical analysis. 

Laboratory tests, when performed, were collected at the time of diagnosis, and 24, 48, 72, 96, 120 and beyond 120 h from diagnosis. Blood count with formula, international normalized ratio (INR), activated partial thromboplastin time (aPTT), D-dimers and fibrinogen values were collected. Data regarding treatments (including steroids, intravenous immunoglobulins—IVIG, anti-coagulant medication, biologic agents), death, hospitalization in the pediatric intensive care unit (PICU), intubation, onset of embolic or hemorrhagic phenomena, non-invasive ventilation, myocardial dysfunction (defined as ejection fraction less than 50%), coronary anomalies (found by performing cardiac imaging) and the presence of sequelae were also collected.

Each center received approval of the local Ethics Committee (ethical committee of Fondazione Policlinico Universitario A Gemelli IRCCS of Rome, prot N. 0015273/20, 06/04/2020, ID 3078; ethical committee of the University of Naples protc 261/20; ethical committee of Costa Rica: CEC-HNN-243-2020; ethical committee of Perù: No. 42-IETSI-ESSALUD-2020). Written informed consent was obtained by patients‘ caregivers.

### Statistical Analyses

For continuous variables the Kolmogorov–Smirnov test was used to assess whether the distribution was normal or not. Categorical variables were reported as count and percentage. Continuous variables with normal distribution were expressed as mean with standard deviation; data with skewed distribution were expressed as median with interquartile range (IQR 25–75%). Statistical comparisons between groups were obtained by Chi-squared tests or Fisher’s exact tests for categorical variables, and Mann–Whitney U-test for continuous variables if not normally distributed. *p* value < 0.05 was considered statistically significant.

In consideration of the low number of deaths that occurred in our case series, the risk factor analysis was performed considering the PICU admission as index of the disease severity and primary outcome. To investigate the role of independent variable as potential risk factors, a univariate logistic regression was performed.

A second univariate logistic regression was performed to investigate the impact of an increase in the value of D-dimers on other outcomes (intubation, onset of embolic phenomena, hemorrhages, non-invasive ventilation, myocardial dysfunction, coronary anomalies and the appearance of sequelae).

Three multivariable logistic regression models were built to evaluate the impact, as an independent variable, of an increase in the value of D-dimers. In consideration of the sample size, we decided to include a maximum number of variables of nine in the multivariable analysis.

The first model was built by inserting the variables D-dimers, age and symptoms at onset; the second model, on the other hand, included D-dimers and the remaining tests at diagnosis; the third model, finally, included D-dimers, rash and possible diagnosis of MIS-C.

The risk was reported as odd ratio and 95% confidence intervals (OR, 95%CI). The continuous variables were standardized to perform the regression analyses, to compare the different OR.

Statistical analysis was performed using IBM SPSS Statistics 23.0 software (IBM Corporation, Armonk, NY, USA).

## 3. Results

### 3.1. Study Population

**Figure 1:**
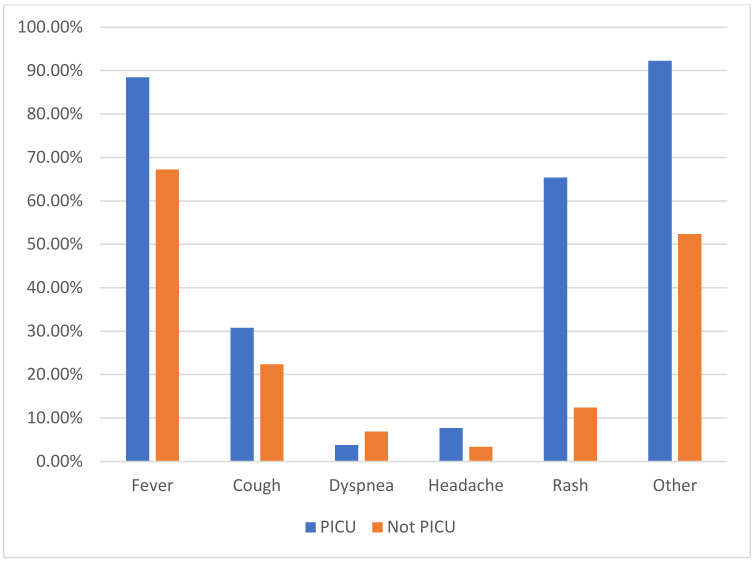
Differences in symptom prevalence between PICU patients and not PICU patients.

Three hundred and sixteen patients (145 females, 45.9%) fulfilled the inclusion criteria and were enrolled in the study, of which 59 (18.7%) received a diagnosis of MIS-C. General characteristics are reported in Table 1 and Figure 1.

The median age was 3.93 years (IQR 0.62–10.7; range 0–17.63 years). Most children (247; 78%) were of Caucasian ethnicity, 13 African (4.1%), 50 Latin American (15.8%) and 6 Asian (1.9%).

Comorbidities were present in 113 (35.8%) children (Supplementary Table S1).

With regard to clinical presentation, 37 patients (11.7%) were asymptomatic, while 218 (69%) had fever, 73 cough (23.1%), 21 dyspnea (6.6%), 12 headaches (3.8%), 53 skin rash (16.8%) and 176 other symptoms (55.7%) (Table 1).

**Figure 2:**
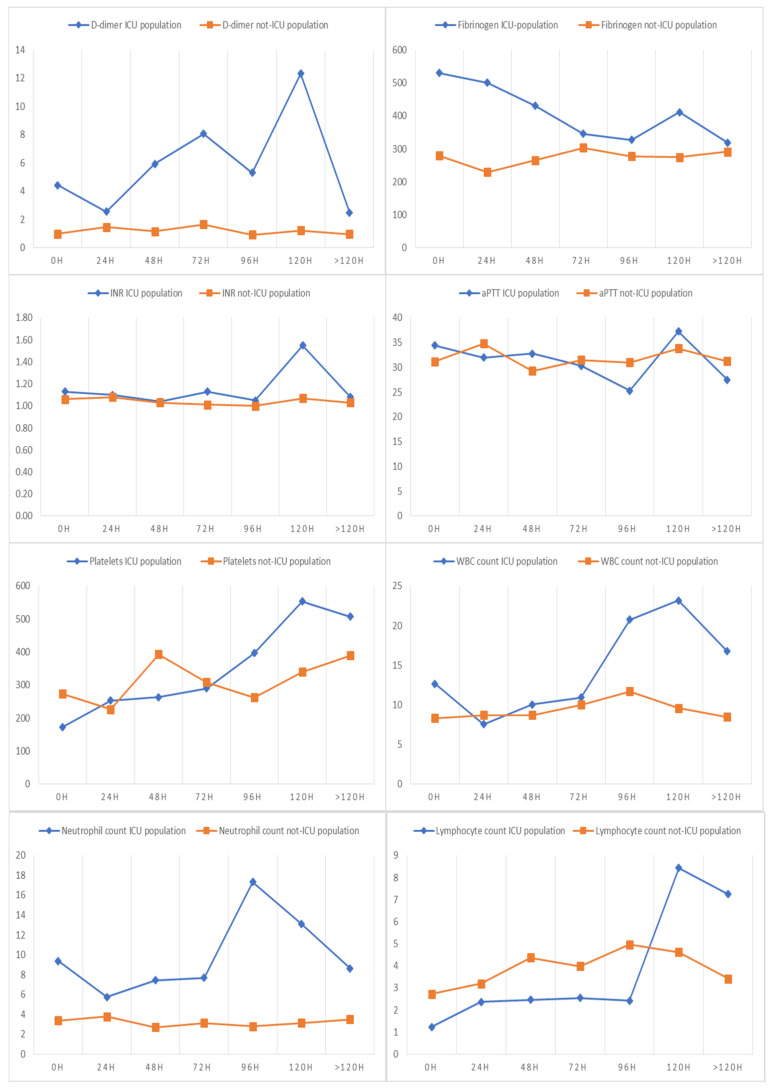
The trend over time of D-dimers (mcg/mL), fibrinogen (mg/dL), aPTT (seconds), INR, platelets (×10^9^ per L), leukocytes (×10^9^ per L), neutrophils (×10^9^ per L) and lymphocytes (×10^9^ per L).

For most patients, it was possible to collect data relating to values of the D-dimers (75.9%), fibrinogen (82.3%), INR and aPTT (74.7%), platelets (86.1%), and leukocyte, neutrophil and lymphocyte counts (80.1%) at diagnosis (Table 1). The data relating to the tests performed at 24, 48, 72, 96, 120 and beyond 120 h are shown in Supplementary Table S2 (A and B panels). The trend over time of D-dimers, fibrinogen, aPTT, INR, platelets, leukocytes, neutrophils and lymphocytes are shown in Figure 2.

Data about treatment received were also collected (Table 2). Specifically, 32 patients (10.1%) received anticoagulant therapy with enoxaparin, 48 (15.2%) aspirin, 56 patients underwent infusion of intravenous immunoglobulin (IVIG) (17.7%) and 30 (9.5%) received other treatments, with the most frequent represented by steroids, antibiotics and supportive therapy, including albumin infusions.

### 3.2. Clinical Outcomes

**Figure 3:**
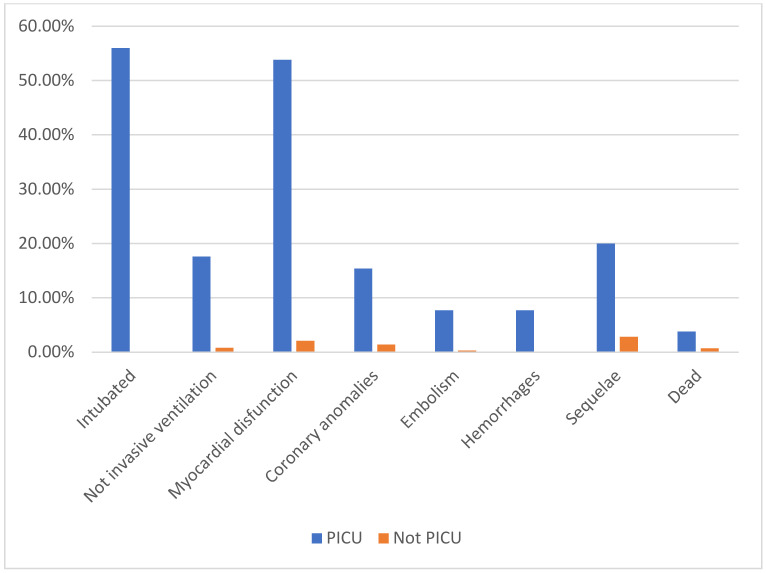
Differences in clinical outcome between PICU patients and not PICU patients.

In our population, 58 patients showed a severe clinical outcome, 13 (4.1%) developed sequelae and 3 (0.9%) died (Table 2 and Figure 3). Specifically, 26 patients (8.2%) were admitted to the PICU, 14 were intubated (4.8%), 5 required non-invasive ventilation (1.8%), 20 developed myocardial dysfunctions (6.3%; in 8 of them (2.5%) coronary anomalies were found), 3 (0.9%) developed an embolic event, 2 (0.6%) had hemorrhages, one patient with liver cancer experienced a hepatic hemorrhage and another a brain event.

### 3.3. Predictors of Disease Severity

The univariate analysis (Table 3) showed that patients with MIS-C, fever, and a skin rash have a higher risk of hospitalization in the PICU. Age, high D-dimer values, hyperfibrinogenemia, INR and aPTT elongation, low platelet count, leukocytosis, high neutrophil values and low lymphocyte values were also associated with an increased risk of PICU admission (Table 3).

With regard to the multivariable analysis, three logistic regression models have been proposed to investigate the real impact of D-dimers on the risk of PICU-hospitalization.

The first model (Table 3), including 183 patients, identified D-dimers at diagnosis as a risk factor independent from age and the other tests performed at admission (OR 1.9, 95%CI 1.11–3.25).

The second model (Table 3), performed on 240 patients, showed that an increase in the value of D-dimers at diagnosis (OR 1.26, 95%CI 1.1–1.44) and the presence of a rash (10.7, 95%CI 3, 5–32.93) are risk factors independent of the presence or absence of other symptoms at the onset.

The third model (Table 4), performed on 240 patients, considers the independent impact of the three variables, that in previous regression analyses had shown the most important associations: the diagnosis of MIS-C, the presence or absence of a rash, and the value of D-dimers. From the latter model it emerged that the diagnosis of MISC-C (OR 12.4, 95% CI 2.3–66.9) and an increase in the value of D-dimers (OR 1.16, 95% CI 1.00–1, 35) are risk factors independent from the presence or absence of a rash.

Table 5 depicts the impact of D-dimer values on other clinical outcomes, showing an association between increased values and a higher risk of PICU admission, intubation and coronary anomalies, myocardial dysfunction and sequelae development.

## 4. Discussion

In this study, we provided a comprehensive clinical and laboratory representation of children with COVID-19 or MIS-C, according to their need of PICU admission. We found that besides MIS-C, D-dimers are an important predictive factor of severe disease and sequelae. To the best of our knowledge, this is the most detailed description of the predictive role of D-dimers in the pediatric population.

With the increase in the number of cases of COVID-19, the need to better understand this pathology appears increasingly urgent; in this sense, identifying risk factors of severity certainly represents one of the most important objectives. As for the general characteristics, our data confirm what has already been reported in the literature, highlighting how older age and the diagnosis of MIS-C are more frequently present in patients admitted to the PICU [21]. On the other hand, there were no statistically significant differences according to gender and the presence or absence of comorbidities, differently from what was reported in other studies [22]. However, it is possible that types of comorbidities were different in the two cohorts and that some specific ones affected outcomes more than others.

From the analysis of the data, we found that fever and a skin rash were the clinical parameters more frequently associated with PICU hospitalization, while higher values of D-dimers, fibrinogen, leukocytes and neutrophils and a reduction in platelets and lymphocytes were the laboratory parameters most likely associated with a worse outcome, in line with available evidence [23]. As expected, the diagnosis of MIS-C was certainly the most relevant risk factor (OR 27.85 with CI 9.91–78.27). The decision to involve patients with two different clinical manifestations of infection (acute SARS-CoV-2 infection and MIS-C) in the analysis stems from the fact that often, especially in the early stages of the disease, the diagnosis of MIS-C may not be immediate and, more importantly, an overlap between severe COVID-19 and MIS-C has been highlighted and their distinction is not always easy, particularly in those children with a positive nasopharyngeal PCR [24]. For this reason, evaluating all patients with a documented SARS-CoV-2 infection, regardless of a confirmed diagnosis of MIS-C, allows us to better define the risk factors and alert practitioners managing children from the moment the infection is detected.

However, the deeper analyses we performed in our study and the regression analyses allowed us to identify several independent risk factors of the severity in children with SARS-CoV-2 or MIS-C, previously never assessed in the literature. The multivariate analysis models were constructed with the aim of evaluating how D-dimers can represent a risk factor for PICU admission, independent of other parameters, including a diagnosis of MIS-C. This is a novel finding since, even in the largest MIS-C study published by the BATS consortium, the role of the D-dimer was not specifically addressed [25]. In the first model, patients’ age and all the laboratory parameters detected at diagnosis were entered. The D-dimers in this model proved to be the only statistically significant independent risk factor (OR 1.9, 95% CI 1.11–3.25), highlighting how this parameter can be more useful than all the other parameters detected at the diagnosis in the risk assessment. This is an important finding since D-dimers are a routine blood test, easy to perform and available in most settings.

The second model was instead built by inserting the value of D-dimers and all the symptoms present at the onset. From this analysis it emerged again that an increase in the value of D-dimers at the onset is an independent risk factor (OR 2.08 with CI 1.36–3.19); however, also the presence of a skin rash (OR 10.7 with CI 3.5–32.93) was reported to be a useful predictive parameter of the severity of the disease. It is possible that a rash has such a predictive role since it is a common manifestation in MIS-C, and, therefore, a rash as part of the MIS-C syndrome has a more important role. In any case, the role of a rash in prediction rules has never been established, and given the easy assessment of this clinical manifestation in outpatient and emergency settings, this still represent a clinically useful finding that can help clinicians working in the front line.

The third model of multivariate analysis was built by inserting the three variables that the previous analyses had shown to have a statistically significant impact: the diagnosis of MIS-C, the presence or absence of a skin rash and an increase in the D-dimer values. It was foreseeable that the diagnosis of MIS-C was associated with a significant increase in the risk of admission to PICU (OR 12.42 with CI 2.3–66.9), independently of the other two variables. Less predictable, although with a less significant OR level (OR 1.61 with CI 1.00–2.59), was the finding that an increase in the value of the D-dimers at diagnosis was also a risk factor independent from the other two variables in determining the risk of PICU admission. Again, these findings have clinical implication for real-world practice in any settings, including in Low-to-Middle Income Countries (LMICs).

A final univariate analysis was conducted to establish the impact of an increased value of D-dimers at diagnosis on the various outcomes analyzed, although these are often poorly represented in our series. As can be highlighted in Table 5, an increase in D-dimers was associated not only with an increased risk of PICU hospitalization, but also with an increased risk of the need for mechanical ventilation or of developing cardiovascular complications or distant sequelae. This observation is also interesting considering the increasing evidence of long COVID-19 (or Post-Acute Sequelae of SARS-CoV-2, PASC) in children [26,27], since adult studies have documented those endothelial abnormalities and microembolisms can play a role in the pathology of PASC, and a recent pediatric study also confirmed the presence of lung perfusion defects months after acute infection [28].

We found no statistically significant associations between the evaluated parameters and the development of embolisms or hemorrhagic events. This is probably due to the low number of children that developed thrombo-embolic phenomena (3/316, about 1% of the study population). This number is in line with the only study that addressed thrombotic events in a large cohort of children in Spain, where 4 patients out of 537 infected children developed a thrombotic complication (prevalence of 0.7% (95% CI: 0.2% to 1.9%) out of the global cohort and 1.1% (95% CI: 0.3% to 2.8%) out of the hospitalized patients) [29]. Furthermore, in this study, the D-dimer value was not specific enough to predict thrombotic complications. However, in the Spanish cohort, D-dimers were available only in 169/537 children (31.5%) and their predictive role was not tested in multivariate analyses.

Altogether, our findings highlight the role of a Covid-induced pro-coagulative inflammatory state that seems to affect the clinical outcomes in children as well, as reported in the adult literature [30]. Recent post-mortem adult studies have clearly documented that lung disease is characterized by “microthrombi”, endothelial inflammation and platelet activation, even in the absence of classic macroscopic embolic events [30]. This condition has now been defined as “thromboinflammation” [31]. Among the most recent established mechanisms, there is evidence that platelet-derived MRP8/14 activates endothelial cells, promotes an inflammatory hypercoagulable phenotype, and is a significant contributor to poor clinical outcomes in COVID-19 patients [32]. Therefore, it is possible to speculate that, even though macroscopic embolic events have rarely been found in our cohort, the laboratory abnormalities we found in our study, and that correlated with poorer outcomes, are biomarkers of an ongoing thromboinflammation, which happens also in children. Not by chance, the most severe spectrum of the SARS-CoV-2 infection in children, such as MIS-C and critical Covid-19, both benefit from anticoagulation. It is still unknown, however, if other children with less severe diseases but evidence of high D-dimer levels would benefit from any treatment or not. This will be an important area of investigation, since abnormal lung perfusion defects have been demonstrated in children even months after recovery from acute infection [28].

Our study has some limitations to address. First, although a relatively large number of children was addressed, relatively few represented the more severe spectrum of disease, limiting our possibility to address other outcomes over PICU admissions. Second, being a multicenter retrospective study, the internal protocols for the management of children with COVID-19 may vary according to the enrolling institutions. In some institutions the coagulation profile was evaluated in all admitted children; in another context, it was performed under medical decisions. Therefore, we may have lost the values of children with a less severe spectrum of COVID-19 assessed in some hospitals or in those rapidly discharged from the emergency department. This is particularly true for children assessed in Latin America, where only those more severely ill have been included in the study, explaining the relatively high rates of complications. Last, PICU admission may also reflect caution by clinicians or administrative requirements rather than disease severity; however, to date the PICU still represents an important outcome addressed in several studies.

In conclusion, our study shows that D-dimers, a widely and easily available test already at first assessment in the emergency departments, play an important role in predicting the more severe spectrum of SARS-CoV-2 infection and, moreover, was higher also in those that developed sequelae, including long COVID-19. These findings support the growing evidence that coagulation and endothelial disease play an important role in acute and long COVID-19 in adults, supporting the need to characterize new biomarkers of endothelial dysfunction in children and their predictive role for acute and long-term sequelae.

## Figures and Tables

**Table 1:** Demographic, clinical and laboratory findings of patients on admission.

	Entire Cohort (n = 316)	PICU (n = 26)	Not PICU (n = 290)	*p* Value
Demographics and clinical characteristics				
Age, years (median, IQR)	3.93 (0.62–10.7)	8 (4.27–12.22)	3.64 (0.43–10.58)	0.005
Sex				0.7
Male, n (%)	171 (54.1%)	15 (57.7%)	156 (53.8%)	
Female, n (%)	145 (45.9%)	11 (42.3%)	134 (46.2%)	
Comorbidities, n (%)	113 (35.8%)	12 (46.2%)	101 (34.8%)	0.24
Signs and symptoms at presentation				
Fever, n (%)	218 (69.0%)	23 (88.5%)	195 (67.2%)	0.02
Cough, n (%)	73 (23.1%)	8 (30.8%)	65 (22.4%)	0.33
Dyspnea, n (%)	21 (6.6%)	1 (3.8%)	20 (6.9%)	1
Headache, n (%)	12 (3.8%)	2 (7.7%)	10 (3.4%)	0.25
Rash, n (%)	53 (16.8%)	17 (65.4%)	36 (12.4%)	<0.0001
Other, n (%)	176 (55.7%)	24 (92.3%)	152 (52.4%)	<0.0001
MIS-C, n (%)	59 (18.7%)	21 (80.8%)	38 (13.1%)	<0.0001
Laboratory findings at the diagnosis				
D-dimer dosage, n (%)	240 (75.9%)	24 (92.3%)	216 (74.5%)	0.05
D-dimer (median, IQR)	1.06 (0.43–2.55)	4.55 (3.38–8.29)	0.99 (0.41–1.78)	<0.0001
D-dimer altered, n (%)	169 (70.4%)	24 (100%)	145 (67.1%)	0.001
Fibrinogen dosage, n (%)	260 (82.3%)	26 (100%)	234 (80.7%)	0.007
Fibrinogen (median, IQR)	286 (233–406)	532 (327–631)	290 (233–438)	<0.0001
Hyperfibrinogenemia, n (%)	65 (25%)	16 (61.5%)	49 (20.9%)	<0.0001
Hypofibrinogenemia, n (%)	28 (10.8%)	3 (11.5%)	25 (10.7%)	0.89
Fibrinogen altered, n (%)	93 (35.8%)	19 (73%)	74 (31.6%)	<0.0001
INR and aPTT dosage, n (%)	236 (74.7%)	26 (100%)	210 (72.4%)	0.001
INR (median, IQR)	1.08 (1–1.17)	1.22 (1.09–1.35)	1.06 (0.98–1.17)	0.003
aPTT, sec (median, IQR)	31.35 (27.58–35)	34 (28.84–42.55)	31.05 (27.4–34.9)	0.31
Platelet count, n (%)	272 (86.1%)	25 (96.2%)	247 (85.2%)	0.15
Platelet count ×10^9^ per L (median, IQR)	268.5 (197.0–345.0)	150.0 (81.0–225.5)	268.5 (205.5–350.5)	0.001
Platelet count altered, n (%)	63 (23.2%)	11 (44.0%)	52 (21.1%)	0.01
Thrombocytopenia, n (%)	39 (14.3%)	9 (36%)	30 (12.1%)	0.001
Thrombocytosis, n (%)	24 (8.8%)	2 (8%)	22 (8.9%)	1
Blood count, n (%)	253 (80.1%)	20 (76.9%)	233 (80.3%)	0.67
White blood cell count, ×10^9^ per L (median, IQR)	8.46 (6.14–12.07)	13.1 (6.4–16.1)	7.9 (5.7–11.5)	0.022
Neutrophil count, ×10^9^ per L (median, IQR)	3.59 (1.79–6.87)	9.4 (5.2–13.0)	3.5 (1.7–6.7)	<0.0001
Lymphocyte count, ×10^9^ per L (median, IQR)	2.59 (1.57–4.64)	1.1 (0.6–1.8)	2.5 (1.5–4.6)	<0.0001

**Table 2:** Treatments and outcomes in the entire cohort and in the intensive care unit children vs. not intensive care unit patients.

	Entire Cohort (n = 316)	PICU (n = 26)	Not PICU (n = 290)	*p* Value
Treatments				
Clexane, n (%)	32 (10.2%)	13 (50%)	19 (6.6%)	<0.0001
Aspirin, n (%)	48 (15.2%)	16 (61.5%)	32 (11.0%)	<0.0001
IVIG, n (%)	56 (17.7%)	20 (76.9%)	36 (12.4%)	<0.0001
Outcomes				
Intubated, n (%)	14 (4.8%)	14 (56.0%)	0 (0%)	<0.0001
Not invasive ventilation, n (%)	5 (1.8%)	3 (17.6%)	2 (0.8%)	0.002
Myocardial disfunction, n (%)	20 (6.3%)	14 (53.8%)	6 (2.1%)	<0.0001
Coronary anomalies, n (%)	8 (2.5%)	4 (15.4%)	4 (1.4%)	0.002
Embolism, n (%)	3 (0.9%)	2 (7.7%)	1 (0.3%)	0.019
Hemorrhages, n (%)	2 (0.6%)	2 (7.7%)	0 (0%)	0.007
Sequelae *, n (%)	13 (4.1%)	5 (20.0%)	8 (2.8%)	<0.0001
Dead, n (%)	3 (0.9%)	1 (3.8%)	2 (0.7%)	0.22

PICU = pediatric intensive care unit. IVIG = intra venous immunoglobulin. * The sequelae observed were: “long COVID” in 3 cases, ileostomy surgery with appendectomy in one case, a delayed administration of anti-lymphocyte serum as treatment of severe bone marrow aplasia due to SARS-CoV-2 infection and successive loss of possibility to receive hematopoietic stem cell transplantation, one case of sacral eschar, two cases of hemophagocytic histiocytosis, an acute pancreatitis, 2 toe necrosis, one necrotizing encephalitis with right hemiparesis, and in one, a patient developed an anxious syndrome.

**Table 3:** Risk factors associated with intensive care unit hospitalization.

	Univariable OR (95% CI)	*p* Value	Multivariable OR (95% CI)	*p* Value	Multivariable OR (95% CI)	*p* Value
**Demographics and clinical characteristics**						
Age, years *	1.56 (1.06–2.3)	0.02	1.1 (0.45–2.65)	0.83		
Male sex (vs. female)	1.1 (0.52–2.63)	0.7				
MIS-C (vs. not-MIS-C)	27.85 (9.91–78.27)	<0.0001				
Comorbidities present (vs. not present)	1.6 (0.71–3.6)	0.25				
Asymptomatic present (vs. not present)	0.28 (0.04–2.15)	0.22				
Fever present (vs. not present)	3.73 (1.09–12.75)	0.03			0.95 (0.17–5.14)	0.95
Cough present (vs. not present)	1.54 (0.64–3.7)	0.33			1.06 (0.37–3.07)	0.9
Dyspnea present (vs. not present)	0.54 (0.07–4.19)	0.57			1.38 (0.13–14.28)	0.79
Headache present (vs. not present)	2.33 (0.48–11.26)	0.29			1.92 (0.27–13.65)	0.51
Rash present (vs. not present)	13.3 (5.52–32.13)	<0.0001			10.7 (3.5–32.93)	<0.0001
Other symptoms ^1^ present (vs. not present)	10.9 (2.52–46.95)	0.001				
**Laboratory findings**						
D-dimer, mcg/mL *	2.67 (1.68–4.23)	<0.0001	1.9 (1.11–3.25)	0.02	2.08 (1.36–3.19)	0.001
Fibrinogen, mg/dL *	1.97 (1.4–2.8)	<0.0001	1.09 (0.65–1.82)	0.74		
Hyperfibrinogenemia present (vs. not present)	6.04 (2.58–14.14)	<0.0001				
INR *	1.51 (1.07–2.12)	0.02	1.24 (0.83–1.85)	0.3		
aPTT, sec *	1.71 (1.21–2.43)	0.002	0.95 (0.58–1.57)	0.85		
Platelet count *	0.47 (0.28–0.77)	0.003	0.6 (0.26–1.37)	0.22		
Thrombocytopenia present (vs. not present)	4.07 (1.65–10.02)	0.002				
White blood cell count *	1.64 (1.13–2.37)	0.008	1.19 (0.13–10.48)	0.88		
Neutrophil count *	1.98 (1.4–2.8)	<0.0001	1.32 (0.15–11.7)	0.8		
Lymphocyte count *	0.08 (0.02–0.33)	<0.0001	0.21 (0.02–1.77)	0.15		

OR = odds ratio. MIS-C = Multisystem inflammatory syndrome in children. * Per 1 standard deviation increase. ^1^ Asthenia, abdominal pain, lack of appetite.

**Table 4:** Risk factors associated with intensive care unit hospitalization.

	Univariable OR (95% CI)	*p* Value	Multivariable OR (95% CI)	*p* Value
**Demographics and clinical characteristics**				
MIS-C (vs. not-MIS-C)	27.85 (9.91–78.27)	<0.0001	12.42 (2.3–66.9)	0.003
Rash present (vs. not present)	13.3 (5.52–32.13)	<0.0001	2.27 (0.57–8.99)	0.241
**Laboratory findings**				
D-dimer, mcg/mL *	2.67 (1.68–4.23)	<0.0001	1.61 (1.00–2.59)	0.046

OR = odds ratio. MIS-C = Multisystem inflammatory syndrome in children. * Per 1 standard deviation increase.

**Table 5:** The impact of a one-unit increase in D-dimer level on different outcomes.

	Univariable OR (95% CI)	*p* Value
PICU admission present (vs. not present)	1.36 (1.17–1.58)	<0.001
Embolism present (vs. not present)	1.11 (0.95–1.29)	1.29
Hemorrhages present (vs. not present)	1.14 (0.98–1.32)	0.07
Intubation present (vs. not present)	1.24 (1.08–1.43)	0.002
Non-invasive ventilation present (vs. not present)	1.13 (0.99–1.29)	0.05
Coronary anomalies present (vs. not present)	1.26 (1.093–1.47)	0.002
Myocardial dysfunction present (vs. not present)	1.27 (1.11–1.46)	<0.001
Sequelae (vs. not present)	1.12 (1.01–1.24)	0.03

OR = odds ratio. MIS-C = Multisystem inflammatory syndrome in children.

## Data Availability

The datasets used and/or analyzed during the current study are available from the corresponding author on reasonable request.

## Supplementary Materials

The following supporting information can be downloaded at: https://www.mdpi.com/article/10.3390/children9020279/s1: Table S1: comorbidities in the study population, Table S2: laboratory exams at 24, 48 and 72 h from the diagnosis, Table S3: laboratory exams at 96, 120 and more than 120 h from the diagnosis.

## References

[1] McCloskey B., Heymann D.L. (2020). SARS to novel coronavirus—Old lessons and new lessons. Epidemiol. Infect..

[2] WHO Coronavirus Disease 2019 (COVID-19) Situation Report. https://covid19.who.int/.

[3] Noh J.Y., Yoon J.G., Seong H., Choi W.S., Sohn J.W., Cheong H.J., Kim W.J., Song J.Y. (2020). Asymptomatic infection and atypical manifestations of COVID-19: Comparison of viral shedding duration. J. Infect..

[4] Noh J.Y., Yoon J.G., Seong H., Choi W.S., Sohn J.W., Cheong H.J., Kim W.J., Song J.Y. (2020). Risk factors associated with acute respiratory distress syndrome and death in patients with coro-na- virus disease 2019 pneumonia in Wuhan, China. JAMA Intern. Med..

[5] Huang C., Wang Y., Li X. (2020). Clinical features of patients infected with 2019 novel coronavirus in Wuhan, China. Lancet.

[6] Tang N., Li D., Wang X. (2020). Abnormal coagulation parameters are associated with poor prognosis in patients with novel coronavirus pneumonia. J. Thromb Haemost..

[7] Nalbandian A., Sehgal K., Gupta A., Madhavan M.V., McGroder C., Stevens J.S., Cook J.R., Nordvig A.S., Shalev D., Sehrawat T.S. (2021). Post-acute COVID-19 syndrome. Nat. Med..

[8] Alsohime F., Temsah M.-H., Al-Nemri A.M., Somily A.M., Al-Subaie S. (2020). COVID-19 infection prevalence in pediatric population: Etiology, clinical presentation, and outcome. J. Infect. Public Heal..

[9] Chen Z.M., Fu J.F., Shu Q., Chen Y.H., Hua C.Z., Li F.B., Lin R., Tang L.-F., Wand T.-L., Wand W. (2020). Diagnosis and treatment recommendations for pediatric respiratory infection caused by the 2019 novel coronavirus. World J. Pediatr..

[10] Stokes E.K., Zambrano L.D., Anderson K.N. (2020). Coronavirus disease 2019 case surveillance—United States, January 22–May 30, 2020. MMWR Morb. Mortal. Wkly Rep..

[11] Gonzalez-Dambrauskas S., Vasquez-Hoyos P., Camporesi A., Cantillano E.M., Dallefeld S., Dominguez-Rojas J., Francoeur C., Gurbanov A., Vega L.M., Shein S. (2021). Adriana Yock-Corrales, ToddKarsies. medRxiv.

[12] Radia T., Williams N., Agrawal P., Harman K., Weale J., Cook J., Gupta A. (2020). Multi-system inflammatory syndrome in children & adolescents (MIS-C): A systematic review of clinical features and presentation. Paediatr. Respir. Rev..

[13] Yasuhara J., Kuno T., Takagi H., Sumitomo N. (2020). Clinical characteristics of COVID-19 in children: A systematic review. Pediatr. Pulmonol..

[14] Wang Y., Zhu F., Wang C., Wu J., Liu J., Chen X., Xiao H., Liu Z., Wu Z., Lu X. (2020). Children Hospitalized with Severe COVID-19 in Wuhan. Pediatr. Infect. Dis. J..

[15] World Health Organization Multisystem inflammatory syndrome in children and adolescents with COVID-19: Scientific brief. 15 May 2020. WHO/2019-nCoV/Sci_Brief/Multisystem_Syndrome_Children/2020.1. https://apps.who.int/iris/handle/10665/332095.

[16] Yock-Corrales A., Lenzi J., Brizuela M., Valentini P., Buonsenso D., COVID-DOMINGO Study Group (2021). Tackling antibiotic resistance during the COVID-19 pandemic is a new challenge for paediatricians. Acta Paediatr..

[17] Yock-Corrales A., Lenzi J., Ulloa-Gutiérrez R., Gómez-Vargas J., Antúnez-Montes O.Y., Aida J.A.R., del Aguila O., Arteaga-Menchaca E., Campos F., Uribe F. (2021). Acute Abdomen and Appendicitis in 1010 Pediatric Patients With COVID-19 or MIS-C: A Multinational Experience from Latin America. Pediatr. Infect. Dis. J..

[18] Yock-Corrales A., Lenzi J., Ulloa-Gutiérrez R., Gómez-Vargas J., Antúnez-Montes O.Y., Aida J.A.R., del Aguila O., Arteaga-Menchaca E., Campos F., Uribe F. (2021). High rates of antibiotic prescriptions in children with COVID-19 or multisystem inflammatory syndrome: A multinational experience in 990 cases from Latin America. Acta Paediatr..

[19] Antúnez-Montes O.Y., Escamilla M.I., Figueroa-Uribe A.F., Arteaga-Menchaca E., Lavariega-Sárachaga M., Salcedo-Lozada P., Sunohara R.A., Melchior P., del Razo J.O.F., Tirado-Caballero J.C. (2020). COVID-19 in South American Children: A Call for Action. Pediatr. Infect. Dis. J..

[20] Antúnez-Montes O.Y., Escamilla M.I., Figueroa-Uribe A.F., Arteaga-Menchaca E., Lavariega-Saráchaga M., Salcedo-Lozada P., Melchior P., de Oliveira R.B., Caballero J.C.T., Redondo H.P. (2020). COVID-19 and Multisystem Inflammatory Syndrome in Latin American Children. Pediatr. Infect. Dis. J..

[21] Martin B., DeWitt P.E., Russell S., Anand A., Bradwell K.R., Bremer C., Gabriel D., Girvin A.T., Hajagos J.G. (2021). Children with SARS-CoV-2 in the National COVID Cohort Collaborative (N3C). medRxiv.

[22] Preston L.E., Chevinsky J.R., Kompaniyets L., Lavery A.M., Kimball A., Boehmer T.K., Goodman A.B. (2021). Characteristics and Disease Severity of US Children and Adolescents Diagnosed With COVID-19. JAMA Netw. Open.

[23] Kara A.A., Böncüoğlu E., Kıymet E., Arıkan K., Şahinkaya S., Düzgöl M., Cem E., Çelebi M., Ağın H., Bayram S.N. (2021). Evaluation of predictors of severe-moderate COVID-19 infections at children: A review of 292 children. J. Med. Virol..

[24] Geva A., Patel M.M., Newhams M.M., Young C.C., Son M.B.F., Kong M., Maddux A.B., Hall M.W., Riggs B.J., Singh A.R. (2021). Data-driven clustering identifies features distinguishing multisystem inflammatory syndrome from acute COVID-19 in children and adolescents. eClinicalMedicine.

[25] McArdle A.J., Vito O., Patel H., Seaby E.G., Shah P., Wilson C., Broderick C., Nijman R., Tremoulet A.H., Munblit D. (2021). Treatment of Multisystem Inflammatory Syndrome in Children. N. Engl. J. Med..

[26] Buonsenso D., Munblit D., De Rose C., Sinatti D., Ricchiuto A., Carfi A., Valentini P. (2021). Preliminary evidence on long COVID in children. Acta Paediatr..

[27] Osmanov I.M., Spiridonova E., Bobkova P., Gamirova A., Shikhaleva A., Andreeva M., Blyuss O., El-Taravi Y., DunnGalvin A., Comberiati P. (2021). Risk factors for post-COVID-19 condition in previously hospitalised children using the ISARIC Global follow-up protocol: A prospective cohort study. Eur. Respir. J..

[28] Buonsenso D., Di Giuda D., Sigfrid L., Pizzuto D.A., Di Sante G., De Rose C., Lazzareschi I., Sali M., Baldi F., Chieffo D.P.R. (2021). Evidence of lung perfusion defects and ongoing inflammation in an adolescent with post-acute sequelae of SARS-CoV-2 infection. Lancet Child. Adolesc. Health.

[29] Aguilera-Alonso D., Murias S., Garde A.M.-D., Soriano-Arandes A., Pareja M., Otheo E., Moraleda C., Tagarro A., Calvo C. (2021). Prevalence of thrombotic complications in children with SARS-CoV-2. Arch. Dis. Child..

[30] Milross L., Majo J., Cooper N., Kaye P.M., Bayraktar O., Filby A., Fisher A.J. (2021). Post-mortem lung tissue: The fossil record of the pathophysiology and immunopathology of severe COVID-19. Lancet Respir. Med..

[31] Guo L., Rondina M.T. (2019). The Era of Thromboinflammation: Platelets Are Dynamic Sensors and Effector Cells During Infectious Diseases. Front. Immunol..

[32] Barrett T.J., Cornwell M., Myndzar K., Rolling C.C., Xia Y., Drenkova K., Biebuyck A., Fields A.T., Tawil M., Luttrell-Williams E. (2021). Platelets amplify endotheliopathy in COVID-19. Sci. Adv..

