# Supplementary material for: Association between Coagulation Profile and Clinical Outcome in Children with SARS-CoV-2 Infection or MIS-C: A Multicenter Cross-Sectional Study
Source: Children (Basel). 2022 Feb 17;9(2):279. doi: 10.3390/children9020279 (PMC8870084; doi:10.3390/children9020279)
Supplement: Supplementary file 1 [file children-09-00279-s001.zip › children-1530390-supplementary.pdf]

SUPPLEMENTARY MATERIAL

Table S1. Comorbidities in the study population.

COMORBIDITIES	N
Neurological pathologies	12
Hematological pathologies	8
Rheumatological pathologies	2
Asthma	9
Other pulmonary pathologies	9
Metabolically pathologies	3
Cardiological pathologies	6
Diabetes	4
Genetical pathologies	8
Oncological pathologies	10
Obesity	12
Nephro-urological pathologies	4
Otolaryngological pathologies	3
Others	23

Table S2. Laboratory exams at 24, 48 and 72 hours form the diagnosis.

	24h	48h	72h
**D-dimer**			
Yes	21 (6,6%)	40 (12,7%)	25 (7,9%)
**D-dimers dosage**			
Media (DS)			
Median (IQR)	1,49 (0,75-3,19)	1,48 (0,71-4,34)	1,75 (1,11-5,15)
**D-dimers altered**			
Yes	16 (76,2%)	32 (80%)	23 (92%)
**Fibrinogen**			
Yes	19 (6%)	36 (11,4%)	24 (7,6%)
**Fibrinogen**			
Media (DS)	...	...	351,04 (149,68)
Median (IQR)	299 (213-582)	346 (250,25-457)	...
**Hyperfibrinogenemia**			
Yes	8 (42,1%)	13 (36,1%)	8 (33,3%)
**Hypofibrinogenemia**			
Yes	2 (10,5%)	3 (8,3%)	1 (4,2%)
**Fibrinogen altered**			
Yes	10 (52,6%)	16 (44,4%)	9 (37,5%)
**Coagulation exams**			
Yes	20 (6,3%)	34 (10,8%)	18 (5,7%)
**INR**			
Media (DS)	...	...	...
Median (IQR)	1,08 (1,02-1,15)	1,03 (1-1,15)	1,05 (0,93-1,15)
**aPTT**			
Media (DS)	35,41 (7,47)	...	32,92 (6,21)
Median (IQR)	...	30,5 (26,97-34,62)	...
**Platelets**			
Yes	18 (5,7%)	32 (10,1%)	23 (7,3%)

**Platelets' count x10⁹ per L**			
Media (DS)	281,9 (157,5-356,0)	...	...
Median (IQR)	...	301,0 (209,2-453,75)	301,0 (226,0-452,0)
**Platelets altered**			
Yes	5 (27,8%)	13 (40,6%)	6 (26,1%)
**Thrombocytopenia**			
Yes	3 (16,7%)	3 (9,4%)	0 (0%)
**Thrombocytosis**			
Yes	2 (11,1%)	8 (25%)	6 (26,1%)
**Blood cell count**			
Yes	15 (4,7%)	31 (9,8%)	23 (7,3%)
**White blood cell count, x10⁹per L**			
Media (DS)	...	9,99 (4,3)	11,4 (6,34)
Median (IQR)	8,56 (8,08-11,52)	...	...
**Neutrophil count, x10⁹ per L**			
Media (DS)	5,23 (3,42)	...	6,78 (5,45)
Median (IQR)	...	3,23 (1,96-7,54)	...
**Lymphocyte count, x10⁹ per L**			
Media (DS)	3,11 (2,61)	...	4,18 (3,11)
Median (IQR)	...	3,13 (2,24-5,13)	...

Table S3. Laboratory exams at 96, 120 and more than 120 hours form the diagnosis.

	**96h**	**120h**	**>120h**
**D-dimer**			
Yes	17 (5,4%)	15 (4,7%)	39 (12,3%)
**D-dimers dosage**			
Media (DS)	...	...	...
Median (IQR)	1,38 (0,83-5,53)	1,77 (0,7-3,57)	1 (0,44-1,64)
**D-dimers altered**			
Yes	15 (88,2%)	13 (86,7%)	26 (66,7%)
**Fibrinogen**			
Yes	17 (5,4%)	15 (4,7%)	36 (11,4%)
**Fibrinogen dosage**			
Media (DS)	...	348,93 (145,9)	...
Median (IQR)	299 (231-432)	...	292 (236,5-350,25)
**Hyperfibrinogenemia**			
Yes	4 (23,5%)	5 (33,3%)	4 (11,9%)
**Hypofibrinogenemia**			
Yes	0 (0%)	2 (13,3%)	4 (11,9%)
**Fibrinogen altered**			
Yes	4 (23,5%)	7 (46,7%)	8 (22,2%)
**Coagulation exams**			
Yes	14 (4,4%)	15 (4,7%)	38 (12%)
**INR**			
Media (DS)	...	...	...
Median (IQR)	1,01 (0,96-1,15)	1,08 (1,02-1,26)	1,03 (0,99-1,11)
**aPTT**			
Media (DS)	30,52 (4,85)	34,81 (7,18)	...
Median (IQR)	...	...	30,55 (27,3-34,72)
**Platelets**			

Yes	12 (3,8%)	13 (4,1%)	35 (11,1%)
**Platelets' count x10^9 per L**			
Media (DS)	275,25 (132,28)	...	...
Median (IQR)	...	340,0 (234,0-637,5)	392,0 (257,0-591,0)
**Platelets altered**			
Yes	4 (33,3%)	6 (46,2%)	13 (37,1%)
**Thrombocytopenia**			
Yes	3 (25%)	1 (7,7%)	1 (2,9%)
**Thrombocytosis**			
Yes	1 (8,3%)	5 (38,5%)	12 (34,3%)
**Blood cell count**			
Yes	12 (3,8%)	11 (3,5%)	32 (10,1%)
**White blood cell count, x10^{9}per L**			
Media (DS)	12,09 (6,47)	12,67 (9,84)	...
Median (IQR)	...	...	8,73 (6,86-12,14)
**Neutrophil count, x10^9 per L**			
Media (DS)	6,62 (6,68)	...	...
Median (IQR)	...	3,54 (2,05-9,46)	3,50 (2,28-6,66)
**Lymphocyte count, x10^9 per L**			
Media (DS)	4,69 (3,37)	4,93 (3,22)	...
Median (IQR)	...	...	3,42 (1,63-6,21)
